# Pediatric Cholestasis: A Practical Approach to Histological Diagnosis

**DOI:** 10.3390/diagnostics16060878

**Published:** 2026-03-16

**Authors:** Francesca Arienzo, Silvia Vallese, Isabella Giovannoni, Andrea Pietrobattista, Marco Spada, Rita Alaggio, Paola Francalanci

**Affiliations:** 1 Pathology Unit, Bambino Gesù Children’s Hospital, IRCCS, 00165 Rome, Italy; 2 Hepatogastroenterology and Liver Transplant Unit and Medical Genetics Laboratory, Bambino Gesù Children’s Hospital, IRCCS, 00165 Rome, Italy; 3 Division of Hepatobiliopancreatic Surgery, Liver and Kidney Transplantation, Bambino Gesù Children’s Hospital, IRCCS, 00165 Rome, Italy; 4 Department of Medical-Surgical Sciences and Biotechnologies, Sapienza University of Rome, 00185 Rome, Italy

**Keywords:** pediatric cholestasis, liver biopsy, neonatal hepatitis, biliary atresia, intrahepatic bile duct paucity, histopathology, molecular diagnostics

## Abstract

Pediatric (neonatal and infantile) jaundice resulting from underlying cholestasis (caused by conjugated hyperbilirubinemia) is always pathological and requires prompt evaluation. Pediatric cholestasis can be caused by medical or surgical factors and, if left untreated, can lead to irreversible liver damage. Timely recognition of pediatric cholestasis and identification of the underlying etiology are paramount to improve outcomes. The broad spectrum of causes potentially underlying pediatric cholestasis requires a multidisciplinary diagnostic approach, and each aspect must be interpreted in the concomitant clinical picture. A liver biopsy is one component of a complex diagnostic puzzle. However, interpreting a liver biopsy performed on a newborn/infant with conjugated/direct hyperbilirubinemia can be a challenging task, as these biopsies are rarely encountered in general hospitals. The aim of this review is to provide a practical and simplified approach to pediatric cholestasis with examples of real clinical cases we have encountered and discuss key features, both histological and clinical, that can help narrow the differential diagnosis and identify treatable causes.

## 1. Introduction

Cholestasis in neonates and infants represents a significant category of disorders, usually defined by an interruption in bile flow (either intrahepatic or extrahepatic), potentially resulting in conjugated (direct) hyperbilirubinemia [1]. In infants with persistent jaundice, the evaluation of fractionated bilirubin—total, conjugated, and unconjugated—is recommended as the preliminary diagnostic measure after two weeks of life to avoid diagnostic delays [2]. Conjugated hyperbilirubinemia is characterized by direct bilirubin levels exceeding 1 mg/dL (17.1 µmol/L) and necessitates further assessment for cholestatic liver disease [3].

This condition affects about 1 in 2500 newborns and shows a higher prevalence among preterm infants [4]. Biliary atresia (BA), the most common identifiable cause of extrahepatic cholestasis, within the first three months of life, represents approximately one-third of all cases; metabolic, genetic, infectious, and idiopathic diseases are among the possible causes of intrahepatic cholestasis [5].

According to current NASPGHAN/ESPGHAN guidelines, infants with conjugated hyperbilirubinemia should be promptly referred to specialized pediatric hepatology centers for comprehensive evaluation [6]. In routine clinical practice, referral and non-invasive investigations—including laboratory testing, abdominal imaging, and, nowadays, genetics—typically precede invasive diagnostic procedures. However, a liver biopsy may be performed when these parameters are inconclusive or do not allow differentiation between intra- and extrahepatic cholestasis. Furthermore, it is important to emphasize that a liver biopsy allows evaluation of other aspects of liver disease: mainly fibrosis, but also inflammation, necrosis, and architectural changes, all of which provide crucial information about disease severity, stage, and potential prognosis, and may help guide subsequent diagnostic and therapeutic decisions.

The diagnostic evaluation of pediatric cholestasis includes liver biopsy as a part of a multidisciplinary approach. It facilitates the distinction between intrahepatic causes of primarily clinical significance and extrahepatic causes of surgical importance, achieving sensitivities and specificities exceeding 90% [7].

A prompt diagnosis is crucial, particularly for extrahepatic conditions such as BA that require urgent surgical intervention. Kasai portoenterostomy performed within the initial 30–60 days of life significantly improves native liver survival and long-term outcomes; however, a delayed diagnosis is associated with irreversible hepatic damage and accelerated progression to end-stage liver disease [8].

Histopathologic examination is an essential component of the diagnostic workup, enabling the identification of morphology and directing subsequent molecular investigations. Early liver biopsy—especially within the first two months of life—has demonstrated enhanced diagnostic accuracy and facilitated clinical decision-making [9]. However, due to the significant heterogeneity of pediatric cholestatic disorders, histopathological findings must be interpreted in conjunction with clinical, biochemical, imaging, and, increasingly, genetic data, as molecular diagnostics are essential for detecting inherited forms of cholestasis [1]. Among laboratory tests, a simple, inexpensive, and readily available test is the γ-Glutamyl Transpeptidase (GGT) level. The GGT level is the first step in guiding subsequent tests. Normal or low values are more consistent with progressive familial intrahepatic cholestasis (PFIC) and bile acid synthesis defects (BASDs), while high values are more indicative of other pathologies [(e.g., acute liver disease, metabolic disease, or infectious diseases; neonatal sclerosing cholangitis (NSC)] [10].

In optimal clinical settings, pediatric liver biopsies are interpreted by pathologists with specific expertise in hepatopathology within specialized referral centers. However, in routine practice, initial evaluation may occur in non-tertiary institutions where subspecialized expertise is not immediately available. In such contexts, recognition of fundamental histopathological patterns becomes particularly important, both to guide preliminary diagnostic reasoning and to facilitate timely referral to specialized multidisciplinary centers.

This review outlines the histological features of pediatric cholestasis and presents a case-based approach.

## 2. Developmental Anatomy and Pathogenesis

The ductal plate is a temporary structure that covers the branches of the portal vein in an embryo. It is a part of the biliary system inside the liver. During the second trimester of pregnancy, the ductal plate remodels. This process involves the generation of new cells, the selective degradation of old cells, and the preservation of tubular structures that will ultimately constitute the intrahepatic bile ducts. There is a link between abnormal remodelling of the ductal plate and several congenital cholangiopathies, such as BA and ciliopathies [11].

Developmental studies have shown that hepatoblasts, which come from definitive endoderm, are bipotential progenitors that can differentiate into either hepatocytes or cholangiocytes [12]. Single-cell genomic analyses have further validated this developmental plasticity by identifying intermediate hepatobiliary hybrid cells [13].

Three main pathways control the growth of the ductal plate and intrahepatic bile ducts: (i) cholangiocyte differentiation, which is controlled by the Notch (Notch signaling pathway), BMP (bone morphogenetic protein), and TGF-β (transforming growth factor beta) pathways [14]; (ii) proliferation and migration of progenitor cells, which are controlled by the FGF (fibroblast growth factor), retinoic acid, hedgehog signaling pathway, and hippo/Yes-associated protein (hippo/YAP) pathway [13]; (iii) the balance between hepatocytes and cholangiocytes derived from hepatic progenitors is mainly regulated by the Notch, Wnt/β-catenin, TGF-β, and hippo/YAP signaling pathways [15,16].

## 3. Histopathologic Patterns in Pediatric Cholestasis

Histopathological material may derive from percutaneous needle biopsy, intraoperative wedge biopsy (e.g., during Kasai portoenterostomy), or examination of the explanted liver at transplantation. In selected cases, a diagnosis may be established based on clinical, imaging, or genetic findings without the need for diagnostic biopsy.

The diagnostic yield of biopsy is influenced by timing, as early lesions—particularly in BA—may display nonspecific features, and, on the other hand, a late biopsy may display a cirrhotic condition, making surgery useless, if not harmful.

Histological evaluation not only corroborates the diagnosis but also informs clinical or surgical management and offers prognostic insights, particularly concerning the extent of fibrosis and ductular alterations. The standard histological evaluation of a liver biopsy looks at the sample’s quality (it should have at least seven portal tracts) and, in particular, in infants with cholestasis, should focus on several key features, particularly those related to bile duct injury and obstruction, summarized in Table 1 [17].

Other relevant findings include those on inflammation (neutrophilic, mononuclear, or mixed); the distribution of inflammation (peribiliary, portal, or involving bile ducts); aggregates of bile-laden macrophages or Kupffer cells, particularly within portal tracts or periportal areas; and hepatocellular edema and swelling [17].

The routine histochemical stains employed are: reticulin stain (fibrosis progression); periodic acid–Schiff (PAS) and PAS with diastase (PAS-D) (to find cytoplasmic inclusions like α-1-antitrypsin globules and immunoglobulin aggregates); Masson’s trichrome stain (collagen/fibrosis is in the portal, periportal, and sinusoidal areas); and Perl’s Prussian blue (iron deposition, e.g., in conditions like hemochromatosis, hemoglobinopathies, and secondary hemosiderosis) [20,21]. The most common stains used to diagnose liver biopsies are CK7 and CK19 to delineate bile ducts and ductules, facilitating the evaluation of ductular reaction, bile duct scarcity, and the identification of intermediate hepatocytes in chronic cholestatic conditions.

Targeted immunohistochemistry (IHC) has revolutionized the diagnostic efficacy of liver biopsy in neonatal cholestasis by facilitating the direct visualization of bile transporter and junctional proteins in formalin-fixed tissue. In particular, a decrease or loss of canalicular staining for bile salt export pump (BSEP) (ABCB11) and multidrug resistance protein 3 (MDR3) (ABCB4) suggests PFIC2 and PFIC3, respectively, whereas loss of tight junction protein 2 (TJP2) expression by IHC is a sensitive and specific marker of PFIC4 [22]. Furthermore, the evaluation of CK7/CK19 expression, in relation to GGT value, aids in distinguishing low-GGT from high-GGT cholestasis [22].

Based on the combined assessment of routine histology, histochemical specific stains, and immunohistochemical markers, liver biopsies in neonates and infants with cholestasis can be analyzed according to distinct histopathological patterns, each linked to a specific set of conditions (Table 2).

**Figure 1:**
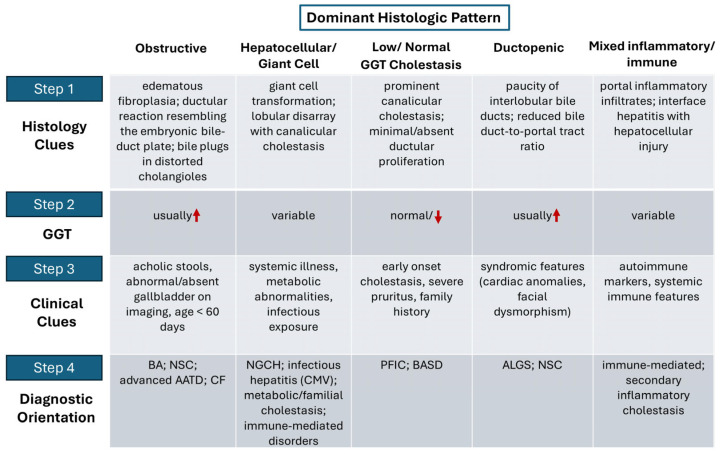
A stepwise clinicopathological integration framework. The upward arrow indicates an increase above the normal range; the downward arrow indicates a decrease. Abbreviations: BA, biliary atresia; NSC, neonatal sclerosing cholangitis; AATD, alpha-1-antitrypsin deficiency; CF, cystic fibrosis; NGCH, neonatal giant cell hepatitis; PFIC, progressive familial intrahepatic cholestasis; BASD, bile acid synthesis defects; ALGS, Alagille syndrome.

To facilitate practical application in routine diagnostic settings, Figure 1 illustrates a stepwise clinicopathological framework that integrates dominant histological patterns with GGT profiles and clinical clues. This algorithmic approach complements the detailed morphologic criteria summarized in Table 1 and the pattern–condition associations outlined in Table 2, providing a structured approach from biopsy interpretation to diagnostic orientation.

This structured approach does not replace guideline-based clinical management but aims to support histopathological reasoning within the broader multidisciplinary diagnostic pathway.

### 3.1. Biliary Atresia

Biliary atresia is the most common identifiable cause of obstructive jaundice within the first three months of life. It is a progressive fibro-inflammatory and obliterative cholangiopathy of infancy, characterized by histological features that change in accordance with the patient’s age and disease progression. There are three classifications of BA: the non-syndromic form (84%); syndromic BA associated with laterality defects (e.g., situs inversus) (10%), and BA accompanied by at least one malformation but without laterality defects (6%). The etiology of BA remains unknown, and proposed theories of pathogenesis encompass genetic factors contributing to bile duct dysmorphogenesis, viral infections, toxins, as well as chronic inflammatory or autoimmune-mediated injury to the bile ducts [6]. A multidisciplinary team comprising pediatricians, hepatologists, radiologists, surgeons, and pathologists establishes a definitive diagnosis of BA.

Sampling peripheral portal tracts in premature infants and infants younger than one month of age can be challenging because the ductal plate remodeling in most of these tracts may not be completed until 4–6 weeks after birth and because bile duct development is directional from the hilar to the peripheral [23]. Therefore, in full-term infants, a liver biopsy should be done between the 40th and 50th day following birth; in premature infants, it should be performed later.

In the early neonatal period (<30 days), liver biopsy may show subtle findings: mild to moderate portal edema with mixed inflammatory infiltrate, diffuse or multifocal ductular reaction, and bile plugs in both larger ducts and peripheral ductules, accompanied by early portal edematous expansion. At more advanced stages (>90 days), marked portal fibrosis with bridging, nodularity, exuberant ductular proliferation, and lobular cholestasis are seen, sometimes with cholestatic hepatocellular injury [24].

Once the diagnosis of BA is established, the primary treatment is a hepatic portoenterostomy (the Kasai procedure), aimed at reestablishing bile flow. When the hepatic portoenterostomy occurs before the age of 60 days, it restores bile excretion in approximately 80% of patients [25]. Yet, in the United States, the age of hepatic portoenterostomy is 63 days (overall median age) and 65 days (overall mean age). However, others have found Kasai portoenterostomy to be beneficial even at older ages [23].

Histological analysis of the porta hepatis in Kasai portoenterostomy specimens generally demonstrates residual bile ductules embedded within fibro-inflammatory stroma, accompanied by reactive epithelium, periductal fibrosis, and myofibroblastic proliferation [24]. Even with successful drainage, most patients will ultimately require liver transplantation [26].

### 3.2. Alpha-1-Antitrypsin Deficiency

The serine protease inhibitor alpha-1-antitrypsin (AAT), which is mostly released by hepatocytes, has a protective antiprotease impact throughout the body, but it is particularly significant in the lung because it can decrease neutrophil elastase activity [27]. The genetic conformational disease known as alpha-1-antitrypsin deficiency (AATD) is caused by a mutant allele that creates a protein that is retained in the hepatocytes’ rough endoplasmic reticulum (RER), where it is synthesized. Its secretion and subsequent accumulation in the hepatocytes are reduced by 85% as a result. The ER-associated degradation process targets the soluble monomers that the aberrant AAT protein generates within the RER, causing them to retrotranslocate into the cytosol and then be degraded by the proteasome machinery [28]. AAT can, however, occasionally form aggregates or polymers within the hepatocyte ER that may lead to liver injury, such as in the Pi*ZZ pathogenic variant [29]. In addition to causing the autophagy process to degrade intrahepatic polymers, the buildup of polymers results in the creation of AAT inclusions, which are visible through PAS staining and persist after diastase digestion (PAS-D) [30]. In certain hepatocytes, the rate of polymerization surpasses the ability for degradation, resulting in AAT inclusions, inflammation, liver cell damage, Kupffer cell activation, and stellate cell induction of fibrosis [31].

AAT mutations cause lung damage, which leads to “genetic emphysema”; liver disease, which manifests as neonatal cholestasis that develops into juvenile cirrhosis; and chronic liver disease, which develops gradually in adults [32]. The primary hereditary cause of liver disease in children is AATD-related. Signs of AATD include newborn cholestasis and elevated levels of direct bilirubin, transaminases, GGT, and serum bile acids in the first few weeks of life in about 10% of Pi*ZZ children [33].

Severe, life-threatening liver disease in children with AATD is infrequent, occurring in approximately 2–3% of cases; however, it may necessitate liver transplantation in the most severe instances. Persistent neonatal cholestasis has been recognized as a significant clinical determinant linked to an unfavorable hepatic prognosis in affected pediatric patients [34]. Transient neonatal cholestasis, mildly elevated liver tests, and hepatomegaly may occur in children; phenotyping and genotyping should be considered when the AAT level is below 1.1 g/L [35].

### 3.3. Bile Acid Synthesis Defects

Bile acid synthesis defects (BASDs) are a heterogeneous group of rare autosomal-recessive disorders caused by defects in enzymes required to convert cholesterol into the primary bile acids, cholic acid and chenodeoxycholic acid. The resulting impairment in bile formation and flow leads to the intracellular accumulation of hepatotoxic intermediates, resulting in a typical picture of cholestasis, usually with normal or low GGT. Although clinical and biochemical findings are essential for diagnosis, liver histology often plays a crucial role in raising suspicion for BASDs and in distinguishing these disorders from other causes of pediatric cholestasis [36].

From a pathophysiological perspective, the absence of primary bile acids disrupts the standard mechanisms of bile-dependent bile flow and the feedback inhibition of bile acid synthesis, resulting in the accumulation of atypical bile acids and bile alcohols capable of damaging hepatocytes, mitochondria, and canalicular structures. This metabolic dysregulation generates histological features that, while not pathognomonic, form a pattern suggestive of BASDs. Canalicular and intracellular cholestasis is commonly observed, typically manifesting as finely granular bile plugs or intracellular bile pigment. Cholestasis is generally less severe than extrahepatic biliary obstruction and is associated with a relatively restricted ductular response [37].

Microvesicular steatosis is one of the most prevalent histological features seen in BASDs. It is hypothesized to be caused by mitochondrial stress and altered lipid metabolism induced by toxic intermediates; hepatocellular ballooning, cytoplasmic clearing, and occasional giant cell transformation may be evident, especially in infants, indicating widespread hepatic damage resulting from metabolic disturbance. These alterations may coincide with early periportal or porto-portal fibrosis, which can advance to cirrhosis if diagnosis and treatment are postponed [36]. BASDs have a lot of the same basic histologic characteristics, although each enzyme deficiency can cause recognizable nuances (Table 3).

The identification of a constellation of characteristics—including low-GGT cholestasis, microvesicular steatosis, mild ductular reaction, and early periportal fibrosis—should necessitate focused biochemical investigations, such as urinary bile acid analysis, in conjunction with confirmatory genetic testing. In this diagnostic framework, liver biopsy is helpful for confirming suspicions of BASDs and evaluating the degree of fibrotic damage, thus offering prognostic insights and aiding therapeutic decision-making, especially concerning the patient’s age. Early diagnosis is crucial, as prompt initiation of primary bile acid supplementation can prevent or even reverse the advancement of liver disease in many BASD subtypes [41].

### 3.4. Progressive Familial Intrahepatic Cholestasis

The histopathological spectrum of progressive familial intrahepatic cholestasis (PFIC) reflects defects in bile canalicular transport proteins, resulting in compromised bile excretion, hepatocellular damage, and advancing fibrosis. While the pathophysiological mechanism differs among subtypes, all forms of PFIC exhibit common histological findings, such as canalicular bile accumulation, hepatocellular ballooning, and portal fibrosis of varying degrees, typically associated with minimal inflammation. As the disease advances, porto-portal bridging fibrosis, lobular architectural distortion, and biliary-type cirrhosis may manifest [42,43]. In the past, the subtype classification was based on IHC for key canalicular transporters, and transmission electron microscopy offered supplementary ultrastructural data in diagnostically difficult cases. The term “PFIC” only meant the three classical types: PFIC1 → *ATP8B1* (FIC1); PFIC2 → *ABCB11* (BSEP); PFIC3 → *ABCB4* (MDR3). Nowadays, the introduction of molecular analysis into the routine diagnostic work-up has expanded the spectrum of classical PFIC, allowing the identification of additional genetic defects that produce phenotypes clinically and histologically consistent with classical PFIC, despite involving distinct molecular mechanisms.

The updated genotype-based classification of PFIC subtypes is summarized in Table 4, adapted from the state-of-the-art review by Vitale et al. [44].

Because PFIC1–3 display reproducible and clinically informative histopathological patterns, they remain central to morphology-based diagnostic algorithms. The recent PFIC subtypes described (gene-drive) 4–13 have overlapping morphological features; they do not add new pattern-based morphological characteristics.

PFIC1 is marked by canalicular cholestasis, a mild ductular reaction, minimal portal inflammation, foamy hepatocytes, and varying levels of fibrosis that may get worse over time [46]. PFIC2 exhibits pronounced canalicular cholestasis with rapid progression to fibrosis and cirrhosis [46]. Notably, PFIC3 presents a cholangiopathic pattern characterized by portal expansion, ductular proliferation, portal inflammation, and bile plugs within interlobular bile ducts, aligning with its elevated GGT profile [47].

Even though all PFIC subtypes have some of the same signs of progressive fibrogenesis, the distribution of fibrosis is different, being predominantly lobular and portal in PFIC1 and PFIC2 and portal-based with bridging fibrosis in PFIC3. Over time, architectural distortion may lead to biliary-type cirrhosis; individuals with PFIC2 have a markedly elevated risk of developing cirrhosis and hepatocellular carcinoma, necessitating vigilant clinical and radiological monitoring throughout childhood and adulthood [44]. From a therapeutic standpoint, the management of PFIC has progressively transitioned towards pharmacological approaches. Medical treatment includes ursodeoxycholic acid for certain subtypes and, more recently, ileal bile acid transporter inhibitors, which have been shown to be effective at lowering serum bile acids, itching, and biochemical cholestasis. These agents may postpone or eliminate the necessity for surgical biliary diversion or liver transplantation in certain patients, especially when administered early in the disease progression [44].

### 3.5. Metabolic Cholestasis

In addition to defects in bile acid synthesis, various congenital metabolic disorders can induce cholestasis in the pediatric population via hepatocellular, mitochondrial, peroxisomal, or direct metabolic toxicity pathways. In suspected cases of metabolic cholestasis, clinical evaluation, biochemical profiling, and genetic testing represent the cornerstone of diagnosis, while liver histology generally plays a supportive rather than definitive role. Although none of the histologic lesions observed in metabolic cholestasis are pathognomonic, the recognition of recurrent histological patterns may assist in narrowing the differential diagnosis and guiding targeted metabolic investigations. Table 5 summarizes the major metabolic and genetic disorders associated with neonatal cholestasis, with emphasis on their characteristic histological features and key diagnostic clues, adapted from the position paper by Ranucci et al. [1].

### 3.6. Infectious Cholestasis: The Role of Cytomegalovirus and Other Pathogens

Infectious etiologies constitute a notable but often overlooked cause of neonatal and infantile cholestasis; infections may arise either congenitally, through transplacental transmission, or postnatally via breast milk or blood transfusion [1,5,48]. Cytomegalovirus (CMV) is the most common infectious cause, followed by other pathogens like Toxoplasma gondii, rubella virus, herpes simplex virus (HSV), enteroviruses, and bacterial infections like Escherichia coli sepsis and Listeria monocytogenes [5,48,49,50,51,52,53,54]. The histologic features are briefly summarized in Table 6.

These infections may cause cholestasis directly by (i) cytopathic injury to hepatocytes and cholangiocytes, (ii) immune-mediated bile duct damage, or (iii) secondary inflammatory changes within the biliary tree [48,54].

In congenital CMV infection, active viral replication in hepatocytes and bile duct epithelial cells results in cholestasis, portal inflammation, and progressive fibrosis [55]. Clinically, CMV-related cholestasis can manifest as isolated neonatal hepatitis, within a multisystem congenital infection, or alongside BA, where CMV is suggested as a potential trigger for immune-mediated bile duct injury [56,57].

CMV-associated cholestasis usually shows up in the first few weeks of life with high direct bilirubin levels, mild hepatomegaly, and high serum transaminases. Serologic testing, polymerase chain reaction (PCR) for CMV DNA, and immunohistochemical detection of viral antigens in liver tissue support the diagnosis [55]. Histologic confirmation is especially beneficial when serologic results are ambiguous or when CMV infection is suspected to act as a cofactor in BA. CMV-positive infants with BA have been documented to exhibit inferior postoperative outcomes subsequent to Kasai portoenterostomy [56,57].

### 3.7. Neonatal Giant Cell Hepatitis

Giant cell transformation is defined by the presence of multinucleated hepatocytes, resulting from either syncytial fusion of adjacent cells or nuclear proliferation without cytokinesis [58]. This phenomenon represents a reactive histopathologic pattern observed across a broad spectrum of neonatal liver disorders, including idiopathic neonatal giant cell hepatitis (NGCH), metabolic cholestasis, infectious hepatitis, and genetic canalicular defects [23]. In idiopathic cases, giant cell transformation may resolve spontaneously over time.

The giant cell hepatitis pattern must always be interpreted in its clinical context, as it may arise from diverse etiologies. Infectious causes, such as CMV, HSV, rubella virus, and enteroviruses, often display associated cytopathic changes or viral inclusions demonstrable by IHC [59,60]. Metabolic cholestasis, including AATD, BASD, and PFIC, may show PAS-D-positive cytoplasmic inclusions or microvesicular steatosis, usually accompanied by mild portal fibrosis [6,61]. Immune-mediated or alloimmune giant cell hepatitis is characterized by prominent lobular inflammation, interface activity, and multifocal hepatocellular necrosis, occasionally mimicking autoimmune hepatitis [2].

Histologically, multinucleated hepatocytes are scattered throughout the lobule, often accentuated in centrilobular zones, with granular or vacuolated cytoplasm and centrally clustered nuclei. The lobular architecture is generally preserved, although mild disarray and focal hepatocellular dropout may be present. Canalicular and hepatocellular cholestasis is frequently observed, with bile plugs in dilated canaliculi and coarse bile pigment within hepatocytes [62]. Despite its distinctive appearance, giant cell transformation represents a nonspecific response to hepatocellular injury rather than a disease entity per se [60]. In idiopathic NGCH, the predominant features include lobular lymphohistiocytic inflammation, Kupffer cell hyperplasia, and spotty hepatocellular necrosis. Portal tracts are typically small, with minimal inflammation and limited bile duct proliferation, features that help distinguish this condition from extrahepatic BA, which is characterized by portal expansion, ductular reaction, and bile ductular plugs [17]. With chronic disease, portal fibrosis may develop, but it lacks the concentric periductal fibrosis typical of obstructive cholangiopathies [63].

In NGCH, idiopathic forms have occasionally been described with a favorable clinical course and spontaneous resolution of cholestasis and associated clinical signs during follow-up, suggesting that in the absence of identifiable underlying causes, spontaneous improvement of liver disease may occur in cholestatic neonates [64].

### 3.8. Immune-Mediated Disorders

Gestational alloimmune liver disease associated with neonatal hemochromatosis (GALD-NH), formerly known as neonatal hemochromatosis, is one of a broad range of conditions that fall under the umbrella of liver disease in early infancy [65]. This condition usually manifests as subacute fetal liver injury and/or congenital cirrhosis. NH and GALD are not interchangeable: While NH is the phenotypic manifestation of severe liver injury in newborns that is most frequently caused by GALD, GALD is a disease or disease process that causes severe fetal liver impairment. Although not inherited, NH seems to be congenital and familial [66]. GALD is mediated by immunoglobulin G (IgG), just like other maternal alloimmune disorders. Beginning around the 12th week of pregnancy, when the neonatal crystallizable fragment receptor is first produced, maternal IgG antibodies are actively transferred from the mother to the fetus across the placenta. When a woman is exposed to a fetal antigen that she does not identify as “self,” she develops gestational alloimmunity [66]. As a result of this exposure, the fetal-derived antigen is sensitized and IgG antibodies are produced [66]. Maternal IgG antibodies against fetal hepatocytes are the cause of GALD. Hepatocyte-specific proteins that are either strongly sequestered in mature liver or exclusively produced by fetal hepatocytes appear to constitute the target antigen [66]. This basic immune activity does not seem to assault extrahepatic tissue or non-hepatocyte liver cells. Hepatocyte lysis and damage to the liver parenchyma result from the activation of the complement classical pathway by the maternal antibody/fetal antigen complex, which also causes the formation of the membrane attack complex, terminal complement cascade, or C5b9 on hepatocytes. Complement-mediated damage to hepatocytes would cause secondary tissue damage, including hepatic and extrahepatic iron excess [67].

The majority of GALD-NH patients exhibit symptoms of fetal disease, and liver damage starts during intrauterine life. During the first few hours to days after delivery, infants frequently have hypoglycemia and exhibit coagulopathy, hypoalbuminemia, jaundice, and edema. Severe damage and a noticeable loss of hepatocytes—sometimes resulting in none at all—are signs of liver pathology [65]. Hepatocytes that survive exhibit canalicular bile plugs, giant cell or pseudoacinar transformation, and coarsely granular siderosis. Cirrhosis and regenerative nodules are frequently seen. There is little inflammation and comparatively little damage to the portal tracts [66]. It is crucial to keep in mind that hepatocyte pathological siderosis is not indicative of NH because it can occur in several newborn liver disorders [67].

In the past, GALD-NH was thought to be a fatal illness. However, the prognosis has improved and the need for liver transplantation has decreased as a result of neonatal therapy with intravenous immunoglobulin and exchange transfusion [65]. GALD-NH is still linked to significant rates of prenatal morbidity and mortality in spite of this approach [65]. A high level of suspicion is still necessary for the diagnosis of GALD-NH.

### 3.9. Alagille Syndrome

The autosomal-dominant, multisystem illness known as Alagille syndrome (ALGS) is caused by a variety of mutations in JAG1 (more than 90%), although a lesser percentage also has mutations in NOTCH2 [68]. Several phenotypic traits within the same family can result from the same genetic mutation [69]. Characteristic facial features, bile duct paucity, chronic cholestasis, and anomalies in the skeletal (butterfly vertebrae), ocular (posterior embryotoxon), renal, vascular, and cardiac (pulmonary stenosis) systems are among the syndrome’s clinical characteristics [69].

The organ traditionally implicated in ALGS is the liver. Cholestasis typically manifests within the first year of life, and many newborns are assessed for conjugated hyperbilirubinemia and scleral icterus within the first few weeks of their birth [70]. Particularly during the first year of life, synthetic liver function is usually preserved. During this time, coagulopathy is probably caused by a fat-soluble vitamin deficiency from severe cholestasis, which results in a vitamin K deficiency that can be readily corrected with supplementation [70]. Intense pruritus, one of the worst symptoms of any cholestatic liver disease, is the most impairing sign of cholestasis in ALGS. It may or may not be linked to jaundice (anicteric pruritus) and is linked to increased serum bile salt levels [70]. The development of cutaneous xanthomas due to hypercholesterolemia is another result of cholestasis [70]. According to reports, only 20–30% of patients with ALGS require a liver transplant, making the hepatic prognosis previously thought to be favorable [70].

Since molecular diagnostics have advanced, liver biopsy is no longer necessary to diagnose ALGS, but it is still a crucial component of clinical diagnosis in the event that molecular testing is not promptly accessible or to distinguish between ALGS and BA [70]. The hallmark of ALGS is still bile duct paucity, which was originally a necessary condition for diagnosis. The interlobular ducts to portal tracts ratio is used to measure it; a ratio of 0.9 to 1.8 is considered normal, and in older infants, a value of less than 0.5 to 0.75 is diagnosed [71]. To determine a correct ratio, it is crucial to have a significant number of assessed portal tracts; currently, 6–10 portal tracts are often adequate using needle biopsies [70]. A giant cell hepatitis pattern, which may be associated with cholestasis and can resemble BA, and sporadic ductular proliferation, typically linked to portal inflammation, represent additional histological features that have been observed.

### 3.10. Neonatal Sclerosing Cholangitis

Neonatal sclerosing cholangitis is a rare and severe neonatal-onset cholestatic liver disease that ultimately progresses to end-stage liver disease, often requiring liver transplantation during childhood [72]. NSC includes both primary and secondary forms and should be considered in the differential diagnosis of prolonged neonatal cholestasis [73].

The main clinical manifestations include jaundice, hepatosplenomegaly, hyperbilirubinemia, and elevated serum GGT, first reported in 1987 [14]. The primary form, which is rare, is represented by genetic NSC associated with mutations in *DCDC2*, involving both intra- and extrahepatic bile ducts and rapidly progressing to biliary cirrhosis [74].

Histologically, NSC is characterized by varying degrees of liver fibrosis, portal tract inflammation, bile duct proliferation or plate malformation (with or without ductal bile plugs), and giant cell changes of hepatocytes. In a significant proportion of cases, biliary cirrhosis develops. Histopathologically, NSC closely resembles BA, requiring biliary imaging for accurate distinction [75].

BA remains the main differential diagnosis in the early stages, whereas primary sclerosing cholangitis is exceptional in the neonatal period. NSC has been associated with two syndromes: (i) Kabuki syndrome, involving facial dysmorphism, developmental delay, growth hormone deficiency, skeletal anomalies, and congenital heart defects [76], and (ii) neonatal ichthyosis-sclerosing cholangitis (NISCH) syndrome, resulting from claudin-1 deficiency [77].

Long-term prognosis depends on the severity of liver disease and, in NISCH, the specific claudin-1 mutation present [77]. Among secondary forms, Langerhans cell histiocytosis is particularly important as it can cause sclerosing cholangiopathy via ductal infiltration [78]. Prolonged parenteral nutrition is another important etiological factor, especially in premature infants, where cholestasis associated with parenteral nutrition can progress to sclerosing cholangiopathy [79].

## 4. Case-Based Diagnostic Approach

Including short clinical cases can help clarify complex aspects of pediatric cholestasis. Real examples show how histological patterns such as ductular reaction, ductopenia, or giant cell change support the differential diagnosis. This practical approach makes it easier for pathologists to apply key criteria in daily practice.

### 4.1. Case 1: Biliary Atresia

Since birth, a four-week-old girl had clay-colored feces and yellowish staining of the eyes. Anemia and abnormal liver parameters [total bilirubin level of 9.18 mg/dL, with a direct bilirubin of 5.9 mg/dL, GGT of 69 U/L, alanine aminotransferase (ALT) of 64 U/L, and aspartate aminotransferase (AST) of 46 U/L] were found during laboratory tests that included liver function tests, a complete blood profile, a coagulation profile, and serum calcium. Additionally, tests for blood urea, creatinine, and serum electrolytes were conducted; all of the results were within the normal range. Cystic fibrosis was ruled out. An intraoperative cholangiogram favored BA, and an abdominal ultrasound revealed a slightly enlarged gallbladder. A Roux-en-Y jejunojejunostomy combined with a modified Kasai porto-enterostomy was performed. An atretic gallbladder, missing hepatic and cystic ducts, and cholestatic liver abnormalities were among the intraoperative findings. A liver biopsy was carried out.

**Figure 2:**
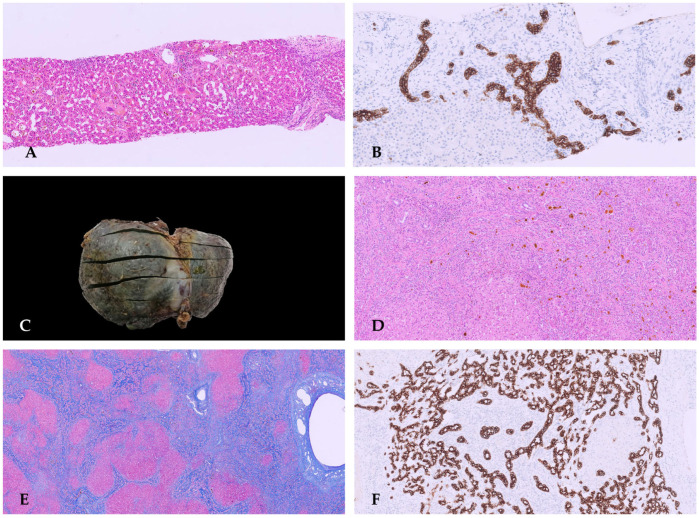
Liver biopsy: hematoxylin and eosin (HE) stain shows altered architecture due to fibrous septum and giant cell/pluri-nucleated hepatocytes (**A**). Immunohistochemistry with CK7 displays dysmorphic bile ductules (**B**). Explanted liver is micronodular and greenish (**C**). Histology: HE shows intrahepatic cholestasis with bile plugs, focal neutrophil infiltration, and focal giant cell transformation (**D**). Masson trichrome stain reveals periportal oedematous fibrous tissue with a cirrhotic pattern (**E**). CK7 indicates a strong ductular reaction reminiscent of a ductal plate malformation (**F**).

Histopathological examination of the biopsy revealed periportal fibro-edema, bile ductular proliferation, intrahepatic cholestasis with bile plugs, focal neutrophil infiltration, ballooning degeneration of hepatocytes, focal giant cell transformation, and EMH consistent with extrahepatic cholestatic disease and fibrosis (Figure 2A,B). These findings were consistent with the diagnosis of extrahepatic BA.

Unfortunately, bile flow was inadequate following the Kasai porto-enterostomy, with rapid development of biliary cirrhosis, portal hypertension, and recurrent cholangitis, and liver transplantation was required 6 months later. Histology of the explanted liver showed biliary cirrhosis (Figure 2C–F).

### 4.2. Case 2: Alpha-1-Antitrypsin Deficiency

**Figure 3:**
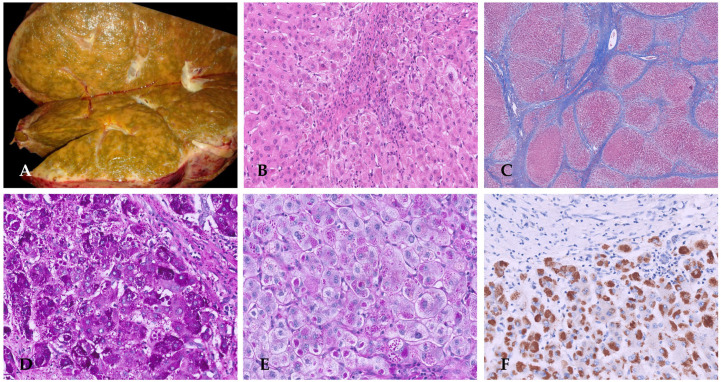
The explanted liver appeared greenish and the surface was greatly deformed by large, protruding nodules separated by deep grooves (**A**). Histology shows portoseptal fibrous expansion with mild ductular reaction, mild inflammation, and diffuse cholestasis (**B**). Masson trichrome staining displays cirrhotic nodules surrounded by a ring of dense collagen and separated by loose fibrous tissue (**C**). PAS (**D**) and PAS-D (**E**) demonstrate the classical findings of diffuse cytoplasmic globules. Immunohistochemical staining for A1AT confirms the accumulation of the abnormal protein (**F**).

A 4-month-old male child presented with neonatal cholestatic jaundice and hepatomegaly. The parents noted multiple soft, seedy, yellow stools daily. Laboratory studies were notable for conjugated hyperbilirubinemia and moderately elevated ALT and AST, with disproportionately elevated GGT. Abdominal ultrasound showed hepatomegaly. The AAT level was 104 mg/dL (normal range 83–199 mg/dL). The AAT genotype indicated a deficiency, specifically the Pi*ZZ phenotype. He was initially treated with supportive care, including ursodeoxycholic acid, but he quickly developed a decompensated disease with worsening ascites and increasing coagulopathy. Documented episodes of bleeding, encephalopathy, pruritus, or dyspnea did not occur. At the age of 3 years, an abdominal ultrasound revealed ascites, cirrhosis, splenomegaly, and reversal of flow in the portal vein, so he was listed for a liver transplantation. In Figure 3, we report the histology of the explanted liver.

### 4.3. Case 3: Bile Acid Synthesis Defects—AKR1D1 Deficiency

A six-month-old boy was born at term, via in vitro fertilization, with a birth weight of 3010 g. Since birth, the patient suffered from jaundice, with ALT/AST values of 936/1547 U/L, an international normalized ratio (INR) of 1.82, and bilirubin total/bilirubin direct values of 13.3/8.65. This condition worsened, leading to hypocolic stools, organ dysfunction, and anemia. The bile acids were recorded at 46.7 µmol/L. On admission ultrasound, the liver was moderately enlarged in size, with rounded margins and a smooth Glissonian profile characterized by inhomogeneous echostructure due to increased parenchymal echogenicity in the absence of focal lesions with expansive characteristics attributable to reactive edema/accumulation with a preserved vascular scaffold. There were no indications of BA. Virological screening was negative, no thyroid disease was detected, lipid and adrenal cortex levels were normal, and AAT, cardiac status, fundus examination, brain ultrasound, and EEG were within the normal range. Metabolic screening was also negative. Genetic analysis showed the presence of a homozygous c.453G>A variant (p.Trp151Ter, nonsense mutation) in the *AKR1D1* gene associated with congenital BASD, type 2. For this reason, therapy with cholic acid was initiated, and coagulation was normalized; bilirubin (minimum bilirubin total/bilirubin direct 6.6/4.6 mg/dL) and transaminase levels were progressively reduced, without ever completely turning negative.

**Figure 4:**
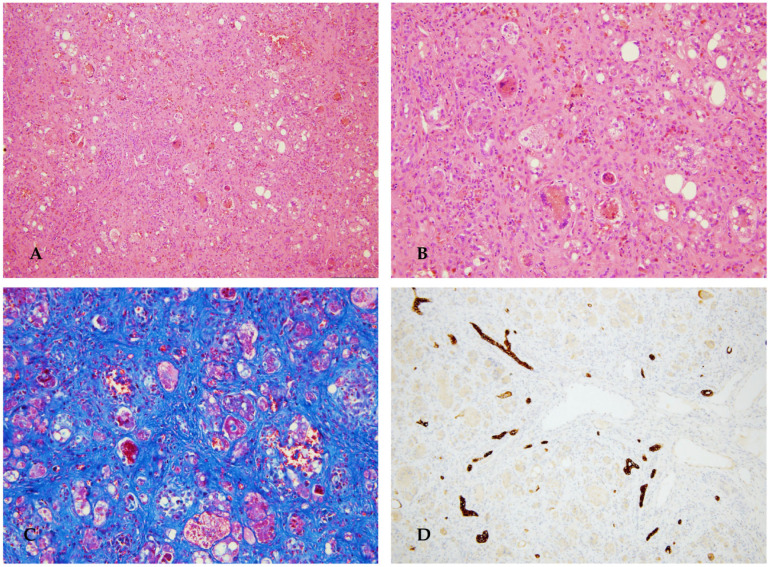
The explanted liver was characterized by severe intralobular cholestasis accompanied by giant cell transformations in hepatocytes (**A**,**B**). Scattered hepatocytes, including giant forms, were degenerate or necrotic. Portal area inflammation included lymphocytes, significant numbers of polymorphonuclear leukocytes, and rare eosinophils. Periportal fibrosis progressed to micronodular cirrhosis (**C**). On IHC, CK7 showed normal biliary ducts (**D**).

The liver condition gradually deteriorated, resulting in coagulopathy (INR 2.2), hypoalbuminemia, elevated cholestasis markers (bilirubin total 13 mg/dL), and anemia (Hb 7.7 g/dL). Blood products for support and the correction of the coagulopathy with plasma and prothrombin complex concentrate were administered. Due to worsening clinical conditions and a pediatric end-stage liver disease score of 33 with a factor V of 37%, the patient was placed on the liver transplant list and underwent a left split transplant after 6 months. The histology of the explanted liver is shown in Figure 4.

### 4.4. Case 4

#### 4.4.1. Case 4.1: Progressive Familial Intrahepatic Cholestasis 2

**Figure 5:**
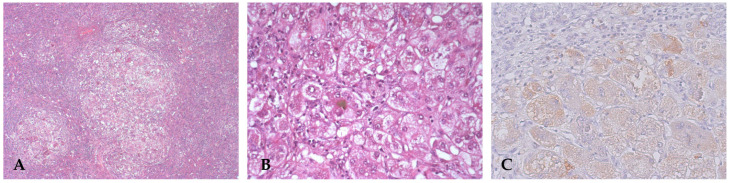
Histology of the patient’s liver tissue reveals advancement of portal fibrosis to micronodular cirrhosis (**A**) and multinucleated large hepatocytes with bile pigment and canalicular cholestasis with the development of cholestatic rosettes (**B**). IHC for BSEP revealed no reaction at all at the hepatocytes’ canalicular pole (**C**).

The patient first presented at 5 months of age with hepatosplenomegaly, pruritus, and jaundice after previously normal growth and development. While GGT levels were normal (38 IU/L), transaminases and serum bilirubin levels were increased (AST 746 IU/L, ALT 546 IU/L, total bilirubin 7.13 mg/dL, and direct bilirubin 5.21 mg/dL). Infectious and metabolic diseases were ruled out, as was a bile acid production deficiency. At the age of nine months, an orthotopic liver transplant was necessary due to deteriorating liver function. A biopsy of the liver was done (Figure 5).

**Figure 6:**
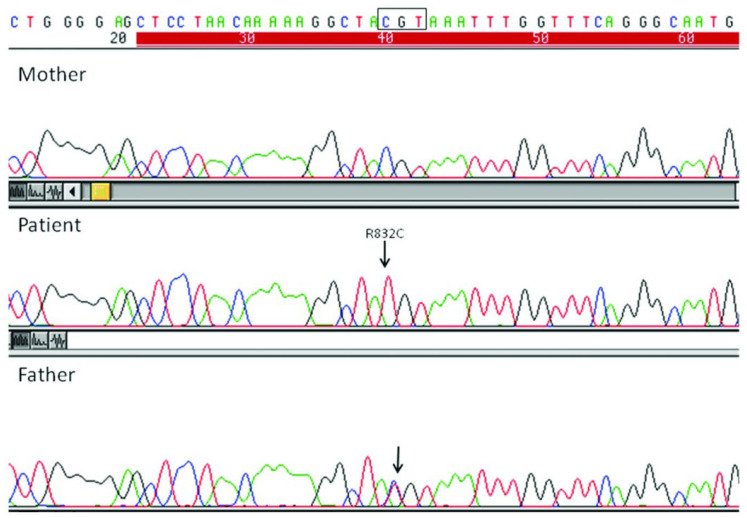
DNA sequence from exon 21 of *ABCB11* gene. The boxed sequence (CGT) indicates the wild-type codon at position 832. DNA analysis shows a normal sequence in the mother, a homozygous c.2494C>T, p.(Arg832Cys) transition (arrow) in the patient, and a heterozygous mutation of the gene in his father.

A homozygous mutation in *ABCB11* exon 21 (c.2494C>T, p.Arg832Cys) linked to PFIC2 was found by molecular studies [80]. The mutation seemed to be homozygous in the patient, heterozygous in his father, and missing in his mother, according to direct gene sequencing (Figure 6).

Somatic mosaicism and the existence of a deletion spanning the maternal copy of *ABCB11* were excluded. Paternal isodisomy is the explanation that most closely matches the data. Microsatellite analysis, however, showed that the infant received the paternal chromosome 2q in a segmental isodisomic manner. Although PFIC2 is caused by an autosomal recessive pattern of transmission, the proband may have inherited two copies of portions of chromosome 2 from his father, according to the molecular genetic data described in this child. This case supports the notion that when homozygosity of an autosomal recessive hereditary mutation occurs, uncomplicated Mendelian inheritance need not necessarily be assumed. In the case of uniparental disomy, the trios’ study may contribute to better genetic counseling [81].

#### 4.4.2. Case 4.2: Progressive Familial Intrahepatic Cholestasis 5

A two-month-old female baby was delivered at 42 weeks of gestation after an uneventful pregnancy. Jaundice was noted when she was 2 months old. On physical examination, she was well-nourished but had icterus and hepatomegaly. Blood tests revealed direct hyperbilirubinemia with total and direct bilirubin of 10.22/5.46 mg/dL, AST/ALT of 81/75 U/L, GGT of 33 U/L, and prolonged INR of up to 3.2.

**Figure 7:**
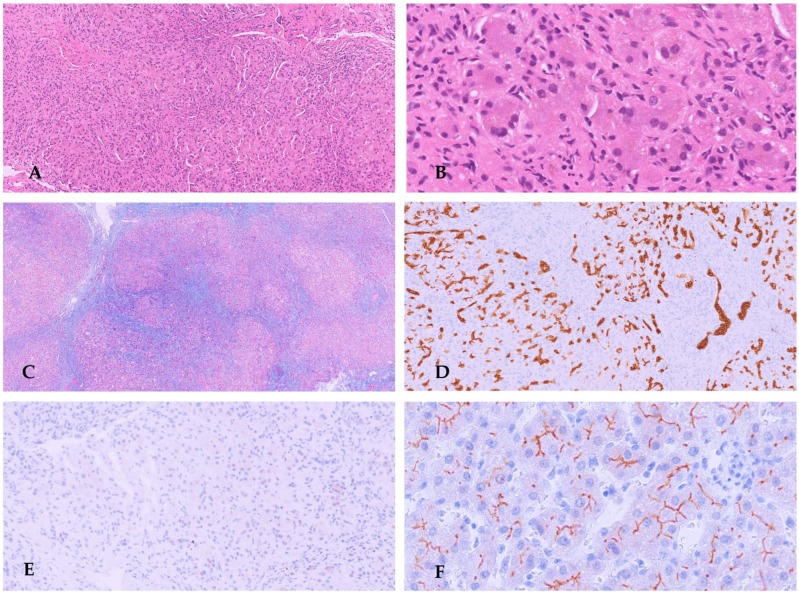
Histology shows severe cholestasis (**A**) and giant cell transformation of the hepatocytes (**B**) with altered architecture due to portal fibrosis with nodular pattern (**C**) and rich ductular proliferation (**D**). IHC showed no detectable expression of BSEP on the canalicular edge of the hepatocytes (**E**), while anti-MDR3 was regularly expressed (**F**), consistent with progressive familial intrahepatic cholestasis type 2 (BSEP deficiency).

A survey for cholestasis, including viral (HSV-1, HSV-2, and CMV) infection and thyroid function, was negative. Abdominal sonography showed coarse liver parenchyma with increased liver stiffness by shear wave elastography. Liver biopsy was performed (Figure 7).

Due to rapidly worsening clinical conditions, with hepatic encephalopathy and hyperammonemia that were poorly responsive to sodium benzoate and bioarginine supplementation, the patient was placed on the liver transplant list due to coagulopathy dependent on plasma and coagulation factor infusions. Liver transplant was performed at 8 months old. A molecular analysis for cholestatic genes (*ATP8B1*, *ABCB11*, *ABCB4*, *TJP2*, *BAAT*, *EPHX1*, *CYP7B1*, *AKR1D1*, *HSD3B7*) showed a homozygous *NR1H4* mutation in exon 8 (c.446G>A, p.Gly149Asp), while no pathological variant was found on *ABCB11* encoding BSEP. A loss-of-function variant in the *NR1H4* gene causes a deficiency of the bile acid receptor known as the FXR. In the liver, FXR is a bile acid-sensitive receptor involved in BSEP expression, and *NR1H4* variants cause loss of BSEP expression on IHC, leading to a misleading diagnosis of BSEP deficiency. Therefore, molecular testing is mandatory for the correct diagnosis of neonatal cholestasis. Thanks to molecular analysis, a final diagnosis of PFIC5 was established.

### 4.5. Case 5: Tyrosinemia Type 1

**Figure 8:**
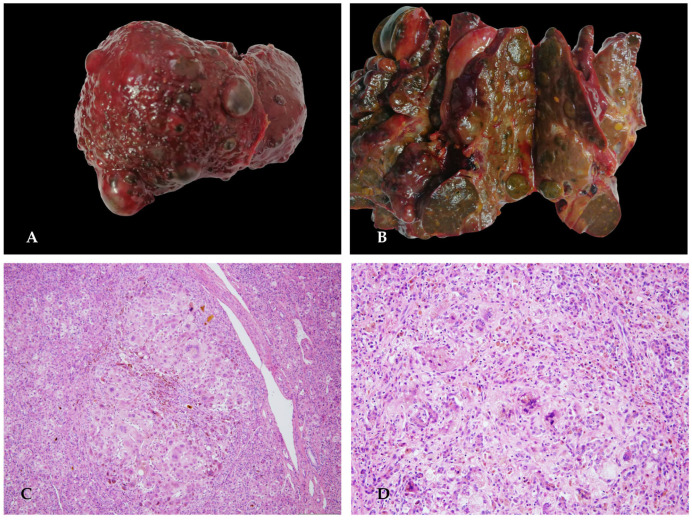
Explanted liver presented a multinodular aspect and greenish color (**A**,**B**). Histological examination showed extensive hepatocyte necrosis and diffuse ductular proliferation with numerous regenerative nodules of various sizes and distinct morphological features: hepatocyte proliferation, sometimes in the form of giant cells with multinucleation and optically empty cytoplasm (cytological dysplasia), intervening foci of extramedullary hematopoiesis, and occasionally small, monomorphic hepatocytes (**C**,**D**).

A firstborn female, born at full term after an uneventful pregnancy, was delivered in the emergency department, with a birth weight of 2700 g. Normal neonatal course. In the first months of life, she was hospitalized for anemia and thrombocytopenia (Hb 8.5 g/dL, PLT 66,000/µL) after 5 days of diarrhea and fever. Positive for COVID serological test. Coagulopathy was already present during this hospitalization (INR 3.9). Then, cholestasis with normal GGT and normal liver enzymes appeared. Radiological investigations (ultrasound plus magnetic resonance) documented a multinodular liver. A liver biopsy was performed, revealing signs compatible with hepatoblastoma. Blood tests confirmed chronic liver disease with hypersplenism (PLT 50,000/µL, Hb 7–9 g/dL, WBC 3500/µL) secondary to splenomegaly from liver fibrosis. Cholestasis with normal GGT (bilirubin total/bilirubin direct 10/6 mg/dL) with mild elevation of AST/ALT (approximately 2–3 times the normal range). The condition was dominated by coagulopathy (INR 4), which required constant blood product supplementation. Elevated alpha-fetoprotein values (above 50,000). Metabolic screening was performed, which indicated a diagnosis of tyrosinemia type 1 (plasma tyrosine 600 µmol/L, succinylacetone positive, homozygous mutation FAH c.1A>G). Treatment with nitisone (1 mg/kg/day, equivalent to 3 mg twice daily) and a diet supplemented with tyrosine-free food were initiated. There was a significant increase in alpha-fetoprotein (147,167 ng/mL). Due to worsening liver function and the strong suspicion of nodules with possible neoplastic transformation, she underwent liver transplantation (Figure 8).

### 4.6. Case 6: Cytomegalovirus Infection

**Figure 9:**
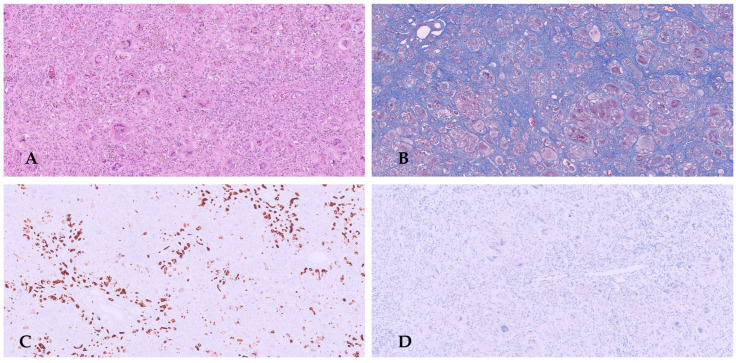
HE slides from the liver showed NGCH with prominent hepatocyte dropout and marked canalicular and cellular cholestasis (**A**). Masson trichrome displays extensive lobular collapse (**B**). Immunhistochemical staining for CK7 indicates significant ductular proliferation (**C**). Many of the remaining hepatocytes showed giant cell transformation. No viral cytopathic change was appreciated, and immunohistochemical staining for CMV was negative (**D**).

At 38 weeks of gestation, a female infant was vaginally delivered. At 6 weeks of gestation, the mother experienced a fever and mild elevation in liver function tests, noted in her prenatal history. According to laboratory analysis, CMV IgM and IgG antibodies were found. Physical examination revealed hepatosplenomegaly and a diffuse petechial and purpuric rash at birth. Severe thrombocytopenia (14,000/µL), direct hyperbilirubinemia (3.2 mg/dL), and high transaminases (ALT, 39 U/L; AST, 233 U/L) were found at the initial laboratory evaluation. CMV PCR of urine was used to confirm CMV. An abdominal ultrasound revealed splenomegaly along with punctate calcifications in the liver and spleen. Although the patient was clinically stable, they initially needed platelet infusions for chronic thrombocytopenia. For symptomatic CMV, ganciclovir treatment was started, and it was later switched to valganciclovir 16 mg/kg/dose, twice daily. However, severe hepatitis with cholestasis and coagulopathy was discovered during a routine check at four months. For treatment, the patient was brought to a local children’s hospital. She was evaluated for a liver transplant. Other contributory viral, toxic, metabolic, or immunological variables were not revealed by additional workup. She developed ascites and her liver failure worsened while she was in the hospital. Intravenous ganciclovir treatment reduced the CMV viral load prior to donation. At the age of 4.5 months, she received a liver transplant utilizing a full left lobe liver allograft that was ABO incompatible. Histology of the liver revealed widespread necrosis (Figure 9).

At 12 months of age, gross motor development was delayed, corresponding to a developmental age of 6–8 months. She remains viremic for CMV and is on valganciclovir.

### 4.7. Case 7: Neonatal Giant Cell Hepatitis

**Figure 10:**
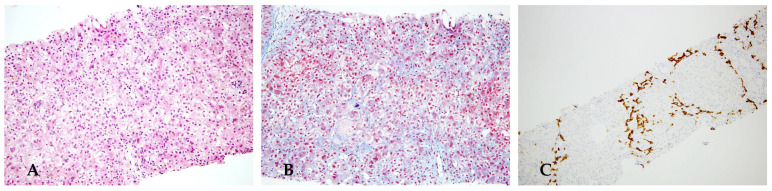
On HE sections, the liver was characterized by cholestasis with some pseudoglandular structures, giant cell transformation, ballooning, apoptotic bodies, EMH, and lobular and portal inflammation (**A**). Masson trichrome displayed mild fibrosis (**B**) and CK7 immunostaining showed ductular proliferation (**C**).

The patient was a male infant, born at 37 weeks and 5 days of gestation by spontaneous vaginal delivery, with Apgar scores of 9 and 10 at 1 and 5 min, respectively. Birth weight was 2600 g. The perinatal course was unremarkable, and the infant was breastfed. At 2 months, jaundice appeared. Upon admission, the patient presented with hyperbilirubinemia and hypertransaminasemia associated with acholic stools, without fever. During hospitalization, laboratory evaluation showed a WBC count of 13,750/mm^3^, Hb 9.2 g/dL, PLTs 150,000/µL, and an INR of 0.97. Total bilirubin was 6.69 mg/dL, with elevated liver enzymes (AST/ALT/GGT: 300/166/530 U/L). Serum creatinine was 0.93 mg/dL. Thyroid function tests were within normal limits (TSH 3.4 mU/mL, free T4 1.56 ng/dL). Alkaline phosphates were elevated (792 U/L), while alpha-1 antitrypsin levels were within the normal range. Hormonal evaluation revealed reduced cortisol levels (2.3 µg/dL) with normal ACTH (27 pg/dL). CMV DNA in urine was negative. Both direct and indirect Coombs tests were negative. Serological testing for Toxoplasma, rubella, CMV, HCV, and HAV was negative, and HBsAg was also negative. Serology showed positive IgG anti-EBV VCA with negative IgM and positive IgG anti-EBNA, consistent with past EBV infection. IgG antibodies against HSV-1/2 were positive, with negative IgM. Abdominal ultrasound revealed a moderately enlarged liver characterized by a heterogeneous echotexture with increased parenchymal echogenicity (reactive edema) and a contracted gallbladder with thickened walls and transonic contents. Due to persistent hyperbilirubinemia, a liver biopsy was performed (Figure 10).

Cardiac, ophthalmological, and audiological screening results were normal. Chest X-ray was normal. Genetic testing for the main forms of cholestasis at this age was negative. Metabolic disease evaluation did not present significant alterations in the tests performed upon admission. Vitamin supplementation and ursodeoxycholic acid therapy were initiated. Subsequently, the jaundice completely resolved, and at 2 years of age, the child shows clinical well-being and eats and grows normally. This case represents an NGCH with a favorable clinical course.

### 4.8. Case 8: Gestational Alloimmune Liver Disease Associated with Neonatal Hemochromatosis

**Figure 11:**
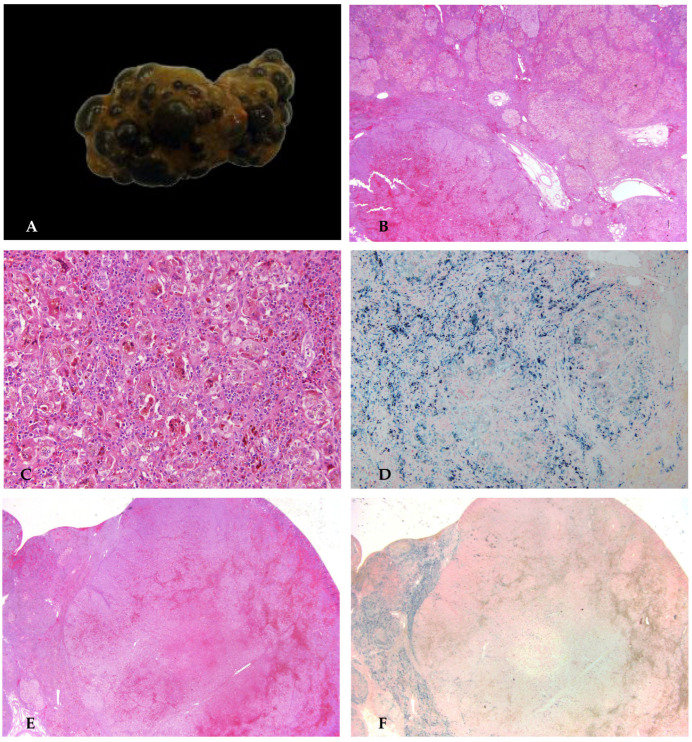
Explanted liver presents micro- and macronodular cirrhosis (**A**). HE sections confirmed altered architecture (**B**), with the residual hepatocytes exhibiting either giant cell or pseudo-acinar transformation (**C**). Perls stain demonstrates diffuse and intense iron deposits in hepatocytes (**D**). Regenerative nodules were present and one macronodule (1 cm in diameter) showed the features of a hepatocellular carcinoma (**E**). Siderosis was more severe in the non-neoplastic liver than in HCC (**F**).

A newborn girl, after 38 weeks and 5 days of gestation, had a birth weight of 1495 g. Parents were nonconsanguineous and the mother had no previous pregnancies and denied any family history of neonatal death or chronic diseases. Soon after birth, she developed hypoglycemia, hyperbilirubinemia, and respiratory distress. She was in profound coagulopathy and required increasing amounts of fresh frozen plasma and factor seven transfusions to maintain a reasonable coagulation profile. Total and direct bilirubin continued to rise (total bilirubin 14.5 mg/dL, direct bilirubin 10.7 mg/dL). AST and ALT were 331 U/L and 76 U/L, respectively. Ferritin was 3014 ng/mL. Alpha-fetoprotein was 99,077.35 ng/mL. Liver ultrasound showed abnormal echo texture with nodular transformation, suggesting cirrhosis. A liver biopsy was performed. Histological examination showed architectural disturbance due to the presence of fibrous septa bordering nodules of mostly giant and multinucleated hepatocytes with feathery degeneration of the cytoplasm. Diffuse bilirubinostasis and multiple foci of extramedullary erythropoiesis were present. Perls staining revealed extensive iron deposits in hepatocytes and biliocytes. She underwent orthotopic liver transplantation at the age of 38 days. The main histological findings of the explanted liver are summarized in Figure 11.

Soon after the reperfusion of the liver, massive abdominal and pulmonary hemorrhages due to intravascular disseminated coagulopathy occurred and the baby died. The explanted liver was small, micro-and macronodular, and deeply bilestained.

### 4.9. Case 9: Alagille Syndrome

**Figure 12:**
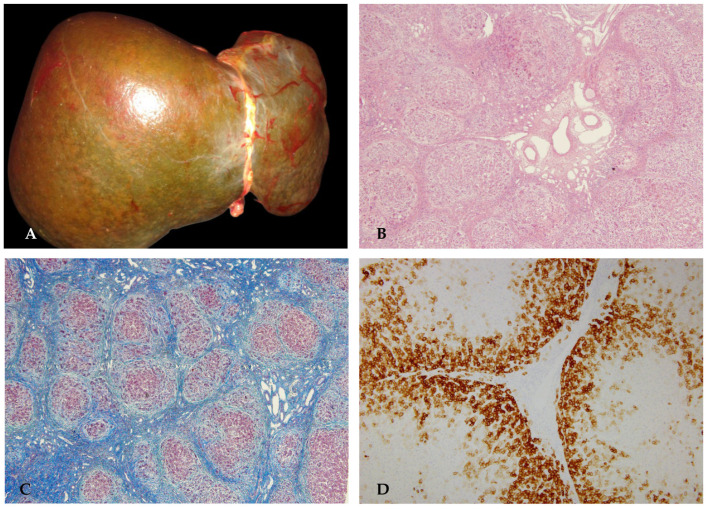
Explanted liver appeared green-brownish and micronodular on the surface (**A**). HE slides showed portal spaces mostly devoid of interlobular bile ducts, with a mild lymphocytic inflammatory infiltrate, excess, dilation, and marginalization of the branches of the portal vein and hepatic artery, with medial hypertrophy of the wall (**B**). Thin fibrous porto-portal septa sometimes arise from portal spaces. Immunohistochemical staining for CK7 confirmed the absence of interlobular bile ducts and displayed widespread phenotypic diversification of hepatocytes, in the absence of ductular reaction (**C**,**D**).

The patient was a full-term male infant, born by spontaneous vaginal delivery, with a birth weight of 2500 g. No significant abnormalities were noted at birth. He was breastfed during the first 3 months of life. The child was admitted to hospital for the presence of jaundice, which appeared after the third month of life. During hospitalization, investigations revealed the presence of butterfly vertebrae and posterior embryotoxon, but no changes on the echocardiogram. Due to persistent jaundice, a liver biopsy was performed; the pathology showed liver parenchyma with preserved architecture, although some thin porto-portal septa were present. In the portal spaces, widened by fibroedema, inflammatory infiltrate, and neoductulogenesis, sometimes, dysmorphic ductules were evident, but, in some portal spaces, the interlobular bile duct was absent. The parenchyma was characterized by bilirubinostasis and giant cell transformation of hepatocytes. These findings raised suspicion of ALGS. Genetic testing revealed a de novo heterozygous p.Pro317Ter mutation in *JAG1*, confirming the clinical diagnosis. The clinical course was characterized by progressive jaundice, the appearance of diffuse xanthelasma, hypercholesterolemia, and persistent and disturbing pruritus. At 3 years of age, a liver transplant was performed (Figure 12).

### 4.10. Case 10: Neonatal Sclerosing Cholangitis

After a normal pregnancy, a little girl weighing 2920 g was born at term. The parents came from the same area but denied consanguinity. Acholic stools and jaundice were observed at two weeks of age. A thorough work-up for newborn cholestasis revealed conjugated hyperbilirubinemia, high GGT, and increased liver enzymes. Septal fibrosis, cholangiolytic alterations, and bilirubin stasis were discovered during a percutaneous liver biopsy. Bilateral nephronophthisis was discovered during the renal evaluation that followed the molecular diagnosis at the age of two. Examinations for hearing and eyesight were normal. Bile duct patency and intrahepatic biliary tree hypoplasia, indicative of NSC, were shown on an intraoperative cholangiogram. The girl experienced progressive portal hypertension in his early years, which led to decompensated liver cirrhosis at age three. In addition, the patient was diagnosed with psychomotor delay and hypotonia. At 12 months, he began to crawl, and at 2 years and 5 months, he began to walk.

She presented a persistent psychomotor delay. Exome sequencing revealed a homozygous variant c.349-2A>G, p.Val117LeufsTer; both parents were asymptomatic carriers. Overall, this case is consistent with NSC due to biallelic DCDC2 mutations, a rare ciliopathy characterized by early-onset cholestasis with high GGT, progressive biliary disease leading to cirrhosis, and multisystem involvement including nephronophthisis and neurodevelopmental delay.

**Figure 13:**
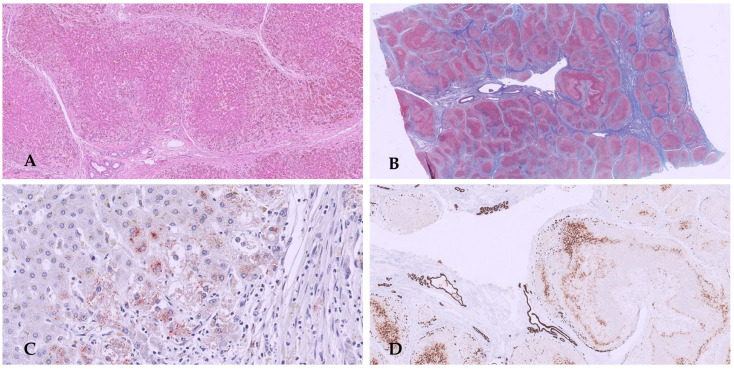
HE section shows disturbed architecture due to numerous fibrous septa encircling hepatocyte nodules (**A**) and diffuse bilirubin stasis. Masson trichrome confirms varying degrees of portal tract fibrosis without oedema (**B**) and a paucity of interlobular bile ducts with occasional intraductal bile in the absence of concentric periductal lamellar fibrosis. Inflammatory cells were very mild, while disarray and atrophy of ductal epithelium were seen focally. Juxtaseptal hepatocytes contained copper deposits (**C**). CK7 immunostaining displays preserved large septal bile ducts and hilar bile ducts with varying dilatation (**D**).

At 15 years old, she underwent liver transplantation. The liver was green and nodular. Histology of the explanted liver showed end-stage biliary cirrhosis (Figure 13).

Unfortunatley due to vascular thrombosis and multiorgan failure, she died after 1 month post-liver transplant.

## 5. Conclusions

In this review, we provided a practical approach to the most common cholestasis in pediatrics. This is a specialized field where liver biopsy is used along with clinical, radiological, laboratory, and, when necessary, molecular information. In cases like BA, quick diagnosis is important to avoid delays in biliary recanalization. BASD, metabolic disorders, and PFIC may exhibit nonspecific or overlapping histological characteristics and should be considered in instances of atypical clinical progression, inconsistent laboratory results, or inconclusive cholangiographic examinations. Infectious cholestatic disease, immune-mediated disorders and neonatal sclerosing cholangitis expand the range of possible differential diagnoses and can resemble BA or other obstructive cholangiopathies at both clinical and histopathological levels. In this intricate diagnostic landscape, meticulous clinicopathological correlation remains essential to guide appropriate management, prognostic assessment, and genetic counseling in pediatric patients with cholestatic liver disease.

## Figures and Tables

**Table 1:** Histological approach to the interpretation of liver biopsies.

Anatomic Domain	Histologic Features	Definition/ Assessment Criteria
Biliary structure and features of obstruction	Bile plugs in bile ducts and/or ductules	Presence of bile plugs within interlobular bile ducts or ductules
	Ductular reaction	Graded as absent, mild, or moderate to marked; further classified as focal (<50% of portal tracts) or generalized (>50%)
	Bile duct proliferation	Number of bile duct profiles per portal tract
Portal architecture	Portal stromal edema	Expansion and clearing of portal stroma
	Interlobular bile duct injury	Cytoplasmic vacuolization of biliary epithelial cells, epithelial necrosis, and loss of cellular polarity
	Portal fibrosis	Staged according to the Scheuer and Batts–Ludwig system (stages 0–4) [18,19]
	DPM	Circumferential arrangement of bile duct-like structures around a central fibrovascular core
Bile duct number	Bile duct paucity	Ratio of interlobular bile ducts to portal tracts ≤ 0.5
Lobular features	Lobular changes	Hepatocellular giant cell transformation, lobular cholestasis, hepatocellular necrosis, lobular inflammation, EMH, macro- or microvesicular steatosis, hepatocellular pseudorosette formation

Abbreviations: DPM, ductal plate malformation; EMH, extramedullary hematopoiesis.

**Table 2:** Histopathologic patterns in neonatal and infantile cholestasis and representative conditions.

Histopathologic Pattern	Main Histological Features	Representative Conditions
Extrahepatic (obstructive) cholestasis	Ductal/ductular bile plugs; marked ductular reaction; bile duct proliferation; portal stromal edema; bridging fibrosis; pseudorosette formation; peribiliary neutrophilic infiltrates	BA; choledochal malformation
Intrahepatic—hepatocellular/lobular (neonatal hepatitis pattern)	Lobular disarray; multinucleated giant cells; hepatocellular cholestasis; macrovesicular steatosis; EMH	Idiopathic neonatal hepatitis; viral/bacterial infections; inborn errors of metabolism; PFIC; parenteral nutrition-associated cholestasis; cystic fibrosis; AATD
Intrahepatic—ductopenic (paucity of interlobular bile ducts)	Reduced bile duct/portal tract ratio; absence or paucity of interlobular bile ducts	ALGS; idiopathic ductopenia; syndromic cholestasis

Abbreviations: BA, biliary atresia; EMH, extramedullary hematopoiesis; PFIC, progressive familial intrahepatic cholestasis; AATD, alpha-1-antitrypsin deficiency; ALGS, Alagille syndrome.

**Table 3:** Histopathologic features in bile acid synthesis defects.

Enzyme Deficiency	Enzyme Mechanism	Main Histological Features
HSD3B7 deficiency	Defect in oxidation and isomerization of 3β-hydroxy-Δ5 bile acids	prominent canalicular and intracellular cholestasis with marked microvesicular steatosis; hepatocytes may contain fine brownish pigment corresponding to accumulated bile acid intermediates; mild ductular reaction and variable periportal fibrosis [38]
AKR1D1 deficiency	Impaired Δ4–3-oxosteroid 5β-reduction	pronounced microvesicular steatosis and ballooning (resembling a pediatric steatohepatitis); cholestasis less conspicuous [39]
AMACR deficiency	Defective peroxisomal β-oxidation of bile acid intermediates	microvesicular steatosis and variable portal fibrosis, indicating a metabolic hepatopathy rather than a cholestatic disorder [39]
CYP7A1 deficiency	Defect in cholesterol 7α-hydroxylation, the first step of bile acid synthesis	a small amount of cholestasis and a lot of macrovesicular steatosis, which is in line with its involvement in the first step of bile acid synthesis ([40,41])

Abbreviations: HSD3B7, 3β-hydroxy-Δ5-C27-steroid oxidoreductase; AKR1D1, aldo-keto reductase family 1 member D1; AMACR, α-methylacyl-CoA racemase; CYP7A1, cholesterol 7α-hydroxylase.

**Table 4:** Classification of PFIC subtypes based on the updated genotype-based findings (adapted from Vitale et al. [44]). The upward arrow indicates an increase above the normal range; the downward arrow indicates a decrease.

PFIC Subtype	Gene (Protein)	Key Histological Features	IHC Profile	GGT	Distinctive Notes
PFIC1	*ATP8B1* (FIC1)	Canalicular cholestasis; foamy hepatocytes; minimal ductular reaction; variable fibrosis	BSEP preserved; MDR3 preserved	N or mildly 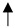	Infantile onset; frequent extrahepatic manifestations (diarrhea, pancreatitis, hearing loss)
PFIC2	*ABCB11* (BSEP)	Marked canalicular cholestasis; hepatocellular injury; minimal inflammation; rapid fibrosis	BSEP absent or markedly reduced; MDR3 preserved	N	High risk of hepatocellular carcinoma; possible recurrence after liver transplantation
PFIC3	*ABCB4* (MDR3)	Portal fibrosis; ductular reaction; bile plugs; portal inflammation; cholangiopathic pattern	MDR3 absent or reduced; BSEP preserved	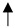	Later onset (childhood/adulthood); overlap with sclerosing cholangitis-like features
PFIC4	*TJP2* (TJP2)	Canalicular cholestasis; mild–moderate portal fibrosis; hepatocellular injury	Claudin-1 reduced or absent; BSEP/MDR3 usually preserved; TPJ2 absent or reduced	N	Early liver failure; increased risk of hepatocellular carcinoma
PFIC5	*NR1H4* (FXR)	Severe neonatal cholestasis; giant cell transformation; early fibrosis	BSEP and MDR3 secondarily reduced	N or 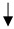	Severe neonatal course; rapidly progressive liver failure
PFIC6	SLC51A OSTα-OSTβ	Variable canalicular cholestasis; minimal inflammation and fibrosis	BSEP reduced or mislocalized	N	Often associated with microvillus inclusion disease; variable intestinal involvement
PFIC7	*USP53* (USP53)	Canalicular cholestasis; mild portal fibrosis	BSEP/MDR3 preserved	N	Frequently associated with sensorineural hearing loss
PFIC8	*KIF12* (Kinesin family member 12)	Ductular reaction; portal fibrosis; cholangiopathic features	Non-specific	N or 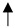	Overlap with biliary tract diseases
PFIC9	*ZFYVE19* (ANCHR)	Cholestasis with variable ductopenia and fibrosis	Non-specific	Variable	Very rare; progressive liver disease reported
PFIC10	*MYO5B* Myosin-Vb	Canalicular cholestasis; hepatocellular injury	Non-specific	N	X-linked inheritance; possible immune dysfunction
PFIC11	SEMA7A Semaphorin-7A	Hepatocellular cholestasis with bile acid accumulation	Non-specific	N or 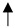	Defective bile acid transport; frequent intestinal involvement
PFIC12	VPS33B vacuolar sorting-associated protein 33B	Cholestasis with giant cell formation	Non-specific	N	Hepatosplenomegaly, mildly prolonged aPTT; arthrogryposis; renal dysfunction–cholestasis (ARC) syndrome
PFIC13	*PSKH1*	Ballooned hepatocytes, cholestasis (bile buildup), giant cells, and fibrosis	Non-specific	N	Hepatorenal ciliopathy [45]

Abbreviations: PFIC, progressive familial intrahepatic cholestasis; BSEP, bile salt export pump; N, normal; MDR3, multidrug resistance protein 3; TJP2, tight junction protein 2; FXR, farnesoid X receptor; GGT, gamma-glutamyl transferase; IHC, immunohistochemistry.

**Table 5:** Metabolic and genetic disorders associated with neonatal cholestasis: histological patterns and diagnostic clues (adapted from Ranucci et al. [1]).

Category	Disorder	Key Histological Features	Notes/Diagnostic Clues
Carbohydrate metabolism	Classic galactosemia	Neonatal cholestatic hepatitis; hepatocellular ballooning; variable macrovesicular steatosis; early portal fibrosis	Detected by newborn screening; elevated galactose/galactose-1-phosphate; GALT deficiency
Amino acid metabolism	Tyrosinemia type 1	Zonal or submassive hepatocellular necrosis; prominent canalicular cholestasis; early bridging fibrosis	Elevated succinylacetone; potentially reversible with nitisinone
Peroxisomal disorders	Zellweger spectrum disorders (PBD)	Diffuse microvesicular steatosis; intracellular cholestasis; paucity or absence of peroxisomes; minimal inflammation	Elevated very-long-chain fatty acids; dysmorphic features; hypotonia
Mitochondrial FA β-oxidation	Fatty acid oxidation disorders (MCAD, LCHAD, TFP)	Diffuse microvesicular steatosis; mild portal inflammation; Reye-like pattern without significant fibrosis	Hypoketotic hypoglycemia; abnormal acylcarnitine profile; episodic metabolic decompensation
Glycogen metabolism	Glycogen storage disease type IV	PAS-positive hepatocytes resistant to diastase; portal fibrosis; rapid progression to cirrhosis	Branching enzyme deficiency; progressive liver failure
Amino acid/urea cycle-related disorder	Citrin deficiency (NICCD)	Mixed macro- and microvesicular steatosis; canalicular cholestasis; ductular reaction; periportal fibrosis	Elevated citrulline; low arginine; prevalent in East Asian populations
Ammonia detoxification defects	Urea cycle disorders	Non-specific changes: patchy hepatocellular necrosis; mild cholestasis; variable steatosis	Severe hyperammonemia; respiratory alkalosis; liver findings may be subtle
Lysosomal storage disorders	Niemann–Pick disease type C	Foamy Kupffer cells; lipid-laden macrophages; variable cholestasis	Elevated oxysterols; hepatosplenomegaly
	Gaucher disease (neonatal form)	Lipid-laden macrophages; portal infiltration; cholestasis	Glucocerebrosidase deficiency; severe neonatal presentation
	Wolman disease	Massive steatosis; adrenal calcifications; foamy histiocytes	Lysosomal acid lipase deficiency; rapidly progressive

Abbreviations: FA, fatty acid; MCAD, medium-chain acyl-CoA dehydrogenase deficiency; LCHAD, long-chain 3-hydroxyacyl-CoA dehydrogenase deficiency; TFP, mitochondrial trifunctional protein deficiency; PBD, peroxisomal biogenesis disorder; PAS, periodic acid–Schiff; GALT, galactose-1-phosphate uridyltransferase; NICCD, neonatal intrahepatic cholestasis caused by citrin deficiency.

**Table 6:** Histopathologic features in congenital/perinatal infectious cholestasis.

Pathogen/Etiology	Main Histological Features	Mechanism of Cholestasis	Diagnostic Clues/Notes
Rubella virus/Congenital infection	Portal fibrosis; bile duct paucity	Developmental ductal injury	Associated with congenital rubella syndrome; systemic anomalies
Herpes simplex virus (HSV)/Congenital or perinatal infections	Diffuse hepatic necrosis; multinucleated giant cells; viral inclusions	Secondary to hepatocellular necrosis	Cholestasis is not primary; often severe systemic illness
Toxoplasma gondii/Congenital infection	Granulomatous hepatitis; portal inflammation	Inflammatory obstruction of bile flow	Often associated with intracranial and ocular findings
Bacterial sepsis (*E. coli*, *Listeria monocytogenes*)/Perinatal infections	Minimal structural ductal damage	Cytokine-mediated inhibition of bile secretion	Cholestasis is functional and reversible with infection control

## Data Availability

No new data were created or analyzed in this study. Data sharing is not applicable to this article.

## Abbreviations

The following abbreviations are used in this manuscript:

AAT	Alpha-1-antitrypsin
AATD	Alpha-1-antitrypsin deficiency
ALGS	Alagille syndrome
ALT	Alanine aminotransferase
AKR1D1	Aldo-keto reductase family 1 member D1
AMACR	α-Methylacyl-CoA racemase
AST	Aspartate aminotransferase
BA	Biliary atresia
BASD	Bile acid synthesis defects
BMP	Bone morphogenetic protein
BSEP	Bile salt export pump
CMV	Cytomegalovirus
CYP7A1	Cholesterol 7α-hydroxylase
DPM	Ductal plate malformation
EMH	Extramedullary hematopoiesis
FA	Fatty acid
FGF	Fibroblast growth factor
FXR	Farnesoid X receptor
GA	Gestational age
GALD-NH	Gestational alloimmune liver disease associated with neonatal hemochromatosis
GALT	Galactose-1-phosphate uridyltransferase
GGT	Gamma-glutamyltransferase
HB	Hemoglobin
HE	Hematoxylin and eosin
Hippo/YAP	Retinoic acid, hedgehog signaling pathway, and hippo/yes-associated protein
HSD3B7	3β-Hydroxy-Δ5-C27-steroid oxidoreductase
HSV	Herpes-simplex virus
IgG	Immunoglobulin G
IHC	Immunohistochemistry
INR	International normalized ratio
IVG	Intravenous immunoglobulin
LCHAD	Long-chain 3-hydroxyacyl-CoA dehydrogenase deficiency
MCAD	Medium-chain acyl-CoA dehydrogenase deficiency
MDR3	Multidrug resistance protein 3
N	Normal
NGCH	Neonatal giant cell hepatitis
NSC	Neonatal sclerosing cholangitis
Notch	Notch signaling pathway
NICCD	Neonatal intrahepatic cholestasis caused by citrin deficiency
NISC	Neonatal ichthyosis-sclerosing cholangitis
PAS	Periodic acid–Schiff
PAS-D	Periodic acid–Schiff with diastase
PBD	Peroxisomal biogenesis disorder
PCR	Polymerase chain reaction
PFIC	Progressive familial intrahepatic cholestasis
PLT	Platelets
RER	Rough endoplasmic reticulum
TFP	Mitochondrial trifunctional protein deficiency
TGF-β	Transforming growth factor beta
TJP2	Tight junction protein 2
WBC	White blood cell
